# Supplementary material for: Each Cellular Compartment Has a Characteristic Protein Reactive Cysteine Ratio Determining Its Sensitivity to Oxidation
Source: Antioxidants (Basel). 2023 Jun 14;12(6):1274. doi: 10.3390/antiox12061274 (PMC10295259; doi:10.3390/antiox12061274)
Supplement: Supplementary file 1 [file antioxidants-12-01274-s001.zip › antioxidants-2343502-supplementary.pdf]

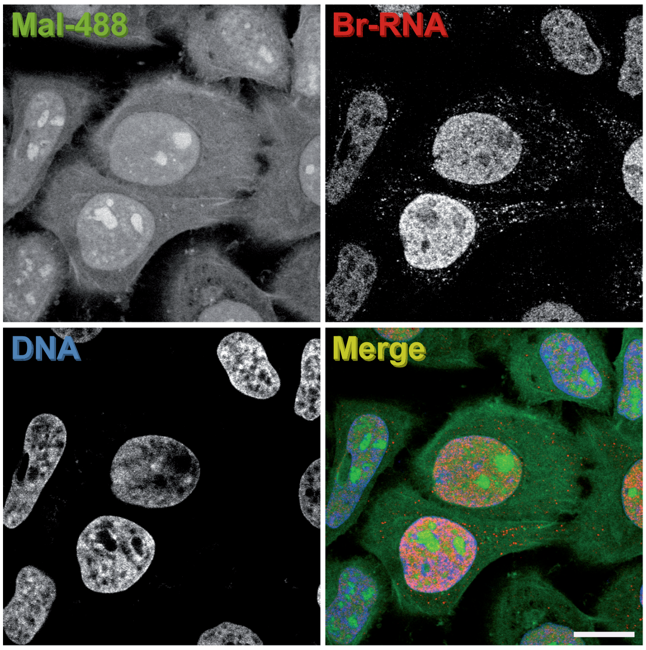

Neves et al., Figure S1

Cellular transcription. The transcriptional activity was measured by the quantification of BrU in-corporation by cells. This figure shows the aspect of BrU incorporated into nuclouplasm, nucleolus, and mitochondria. Images such as this one were used for the quantitative analysis. Bar 5 µm.

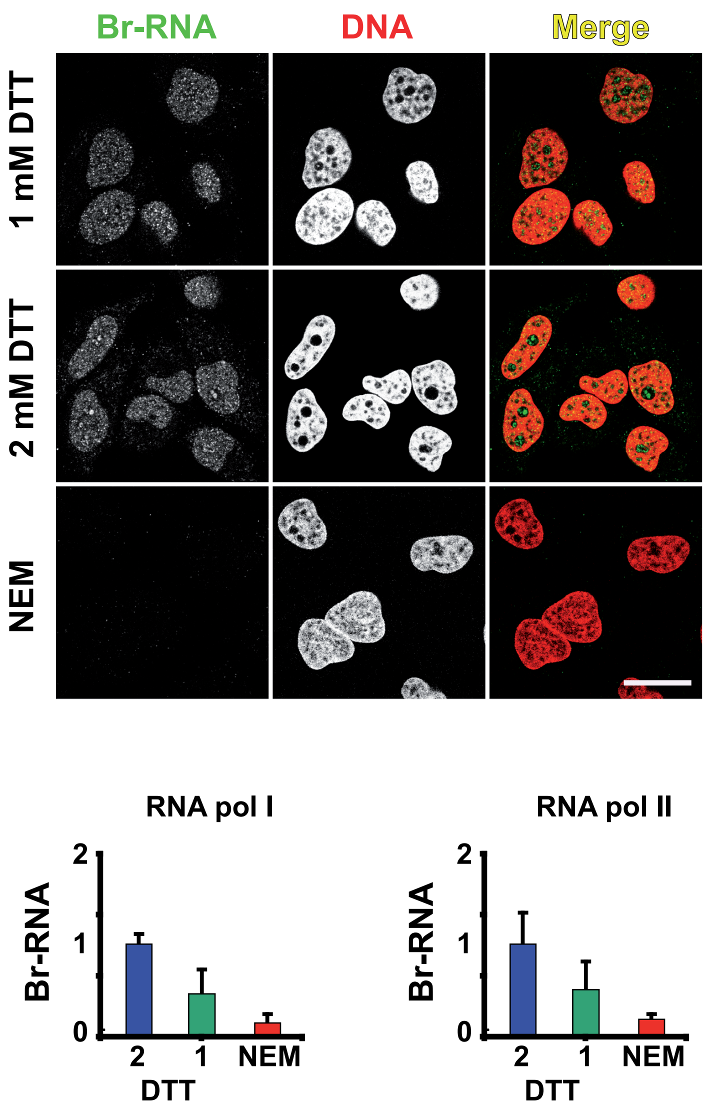

Neves et al., Figure S2

Cellular transcription is sensitive to thiolate content. The transcriptional activity was monitorized by Br-UTP incorporation into nascent RNA. For this panel cells were incubated with 1 or 2 mM DTT in the reaction cocktail or alternatively 1 mM NEM. The increase in DTT resulted in the rise of BrUTP incorporation in both nucleoplasm and nucleolus, conversely, NEM abolished BrUTP in both compartments. The bar diagrams show the quantification of nucleolar signal (RNA pol I) or nucleoplasmic (RNA pol II). Bar 10 µm.

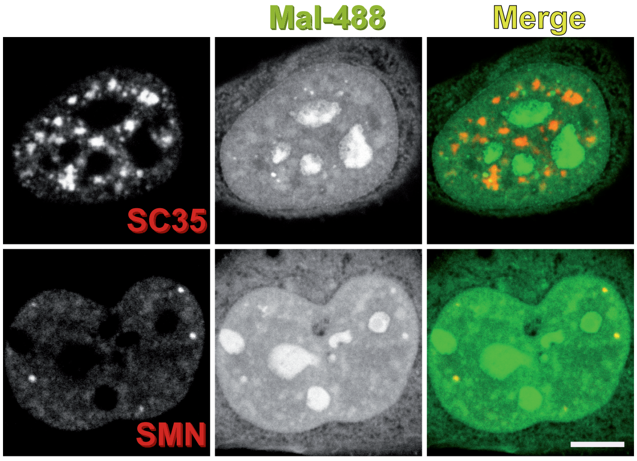

Neves et al., Figure S3

Sub-nuclear distribution of thiolate groups. Apart from the nucleolus Mal-488 concentrate at SC35 nuclear speckles or SMN nuclear bodies. Bar 2 µm.

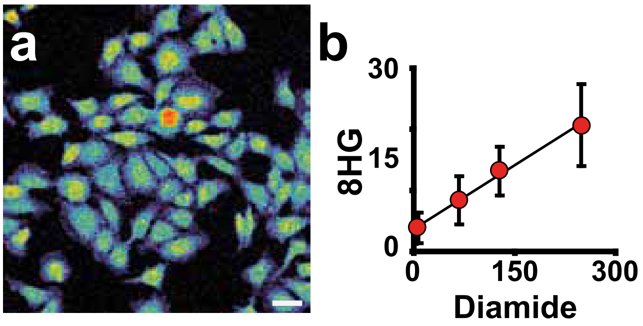

Neves et al., Figure S4

RNA is very sensitive to oxidative stress. In order to determine the sensitivity of RNA to ROS cells were depleted of GSH by diamide exposure for 1 h. (a) The 8HG staining distributes through the entire cell. (b) Quantification of 8HG. The exposure to 250 mM diamide resulted in an approximately 20 times increase in RNA oxidation level. Bar 10 µm.
